# TLR7 Activation of Macrophages by Imiquimod Inhibits HIV Infection through Modulation of Viral Entry Cellular Factors

**DOI:** 10.3390/biology10070661

**Published:** 2021-07-13

**Authors:** Feng-Zhen Meng, Jin-Biao Liu, Xu Wang, Peng Wang, Wen-Hui Hu, Wei Hou, Wen-Zhe Ho

**Affiliations:** 1 School of Basic Medical Sciences, Wuhan University, Wuhan 430000, China; mengfz0128@126.com; 2 Department of Pathology and Laboratory Medicine, Temple University School of Medicine, Philadelphia, PA 19140, USA; jinbiao.liu@temple.edu (J.-B.L.); xu.wang@temple.edu (X.W.); peng.wang@temple.edu (P.W.); whu@temple.edu (W.-H.H.)

**Keywords:** Toll-like receptor 7, CC chemokine, macrophages, human immunodeficiency virus, imiquimod

## Abstract

**Simple Summary:**

The Toll-like receptor (TLR) 7 is highly expressed by immune cells including macrophages. Agonists to TLR7 are attractive therapeutic agents as they have the potential of activating both innate and acquired immunity against viral infections. Imiquimod, a specific TLR7 agonist, has been successfully used for the topical treatment of genital/perianal warts in immunocompetent individuals. Here we examined the anti-HIV effect of imiquimod in primary human macrophages and demonstrated that TLR7 activation by imiquimod could effectively inhibit infection of the cells by different strains of HIV. Further mechanistic studies revealed that while imiquimod had little effect on interferons expression, its treatment of macrophages resulted in the increased production of the CC chemokines, the natural ligands of the HIV entry co-receptor CCR5, and decreased expression of CD4 and CCR5. These findings are clinically important and indicate that activating the intracellular antiviral immunity by imiquimod has the potential for developing TLR7 agonist-based therapy for HIV infection.

**Abstract:**

The Toll-like receptor (TLR) 7 is a viral sensor for detecting single-stranded ribonucleic acid (ssRNA), the activation of which can induce intracellular innate immunity against viral infections. Imiquimod, a synthetic ligand for TLR7, has been successfully used for the topical treatment of genital/perianal warts in immunocompetent individuals. We studied the effect of imiquimod on the human immunodeficiency virus (HIV) infection of primary human macrophages and demonstrated that the treatment of cells with imiquimod effectively inhibited infection with multiple strains (Bal, YU2, and Jago) of HIV. This anti-HIV activity of imiquimod was the most potent when macrophages were treated prior to infection. Infection of macrophages with pseudotyped HIV NL4-3-ΔEnv-eGFP-Bal showed that imiquimod could block the viral entry. Further mechanistic studies revealed that while imiquimod had little effect on the interferons (IFNs) expression, its treatment of macrophages resulted in the increased production of the CC chemokines (human macrophage inflammatory protein-1 alpha (MIP-1α), MIP-1β, and upon activation regulated normal T cells expressed and secreted (RANTES)), the natural ligands of HIV entry co-receptor CCR5, and decreased the expression of CD4 and CCR5. The addition of the antibodies against the CC chemokines to macrophage cultures could block imiquimod-mediated HIV inhibition. These findings provide experimental evidence to support the notion that TLR7 participates in the intracellular immunity against HIV in macrophages, suggesting the further clinical evaluation of imiquimod for its additional benefit of treating genital/perianal warts in people infected with HIV.

## 1. Introduction

Intracellular innate immunity is an essential component of host immune responses against virus infections including HIV [1]. Macrophages play an important role in the immunopathogenesis of HIV infection and the development of acquired immunodeficiency syndrome (AIDS). Macrophages, along with CD4^+^ T cells, are the major targets of HIV [2,3]. Because macrophages express both the primary receptor CD4 and co-receptor CCR5, they are highly susceptible to productive infection by CCR5-tropic HIV strains [4]. It is known that antiretroviral therapy (ART) effectively suppresses HIV in infected individuals. However, even long-term ART could not eradicate HIV in cellular reservoirs, including macrophages [5]. When compared to CD4^+^ T cells, HIV-infected macrophages are more resistant to apoptosis and less sensitive to ART. These pathological properties of macrophages make them an ideal reservoir for HIV persistence and latency as well as a therapeutic target [2,6]. In addition, because of their ability to infiltrate virtually all organs, HIV-infected macrophages contribute to the spread of the virus [3]. Therefore, it is important to target macrophages and identify cellular restriction factors that can suppress and eliminate HIV in these cells.

Innate immunity is composed of several families of pattern recognition receptors (PRRs) that recognize molecular structures of pathogens, known as pathogen-associated molecular patterns (PAMPs) [7]. TLRs are the first PRRs to be identified and the most well-characterized [8,9]. TLRs are largely classified into two subfamilies based on their localization: those on the cell surface and the intracellular endosomal TLRs. TLR7 is one of the intracellular TLRs which recognizes ssRNA. Through TLR7, the host cells detect the invasion of the ssRNA viruses, including HIV [10]. Importantly, the activation of TLR7 signaling pathways results in the release of antiviral and pro-inflammatory cytokines, such as type I interferons (IFN-α and IFN-β) which exert antiviral activities by inducing IFN-stimulated genes (ISGs). Studies have shown that the activation of TLR7/8-mediated signaling pathways upon treatment, with either ssRNA or the small molecule TLR7/8 agonist such as resiquimod (R-848), greatly reduced the ability of lymphoid tissue to support HIV infection [11,12,13]. The investigators concluded that the combined effects of multiple cytokine changes induced by resiquimod, rather than IFN-α, were primarily responsible for the resiquimod-induced antiviral effect [11].

As a synthetic and selective ligand for TLR7, imiquimod can specifically stimulate TLR7 [14] and induce potent antiviral activities against some pathogenic human viruses including herpes simplex virus 2 [15], Sendai virus [16], human papillomavirus (HPV) [17], and influenza A virus [18]. Furthermore, Angelopoulou et al. [19] recently reported that imiquimod can be an ideal option for the management of SARS-CoV-2 infection. Clinically, imiquimod cream has been successfully used for the treatment of external genital and perianal warts in patients infected with both HPV and HIV [17,20,21,22,23] as it stimulates cell-mediated immune responses through the localized induction of IFN-α and other cytokines. Imiquimod is an immunomodulator of monocytes, macrophages, and dendritic cells which express TLR7 and produce IFNs as well as other antiviral cellular factors. However, studies have shown that the ability of these immune cells to produce type I IFNs and ISGs is impaired by HIV infection [24,25,26,27,28]. Liu et al. [29] recently demonstrated that the HIV infection of primary human macrophages and microglial cells suppressed the TLR3 activation-induced expression of the antiviral ISGs and the HIV-restriction miRNAs. While these findings suggest a mechanism for HIV persistence and latency in macrophages and microglial cells, they also indicate the importance and necessity to determine whether to immunologically activate intracellular innate immunity by imiquimod, a TLR7 agonist, to protect macrophages from HIV infection and suppress the viral replication.

## 2. Materials and Methods

### 2.1. Cell Culture

Freshly isolated human peripheral monocytes were obtained from the Human Immunology Core at the University of Pennsylvania. The culture conditions for the monocytes’ differentiation into macrophages were described previously [30,31]. From day 5 to day 7 in culture, the majority (>95%) of the monocytes became macrophages, which showed the characteristic morphology (spindle-shaped and large multinucleated cells) of primary human macrophages while they remained positive for the CD14 marker. HEK-293 T cells were maintained in DMEM (Gibco, New York, NY, USA) with 1% MEM NEAA (Gibco, New York, NY, USA), 1% penicillin-streptomycin (Lonza, Walkersville, GA, USA), 0.5% L-Glutamine (Gibco, New York, NY, USA), and 10% fetal bovine serum (Corning, New York, NY, USA).

### 2.2. Virus and Reagents

Based on the differential usage of co-receptors (CCR5 and CXCR4), HIV isolates have been referred to as R5-, X4-, or dual-tropic strains [32]. The HIV R5-tropic strains (Bal, Jago, and YU2) were obtained from the AIDS Research and Reference Reagent Program of the National Institute of Health (NIH, Bethesda, Rockville, MD, USA). Imiquimod, a synthetic TLR7 ligand, was purchased from InvivoGen (San Diego, CA, USA). MIP-1α, MIP-1β, and RANTES antibodies were purchased from R&D System (R&D system Inc., Minneapolis, MN, USA). Mouse IgG was purchased from Sigma-Aldrich (St. Louis, MO, USA). Rabbit antibody against glyceraldehyde 3-phosphate dehydrogenase (GAPDH), anti-mouse IgG (horseradish peroxidase (HRP)-linked) antibody, and anti-rabbit IgG (HRP-linked) antibody were purchased from Cell Signaling Technology (Danvers, MA, USA). Mouse antibody against HIV-1 p24 was purchased from Abcam (Abcam, Cambridge, UK). All antibodies and reagents for flow cytometry assay were purchased from BD Bioscience (BD Bioscience, San Jose, CA, USA). Trichloroacetic acid (TCA) and acetone were purchased from Sigma-Aldrich.

### 2.3. Preparation of Virus Stocks

pHIV R3A-HSA was kindly provided by Dr. Lishan Su (Department of Microbiology and Immunology, The Lineberger Comprehensive Cancer Center, School of Medicine, The University of North Carolina). This is an HIV molecular clone with a highly pathogenic dual-tropic envelope (R3A) in the NL4-3 backbone [33], which was constructed by replacing the *vpr* gene with a mouse heat-stable antigen (HSA; CD24) [34]. The env defective HIV NL4-3 derivative pHIV NL4-3-ΔEnv-eGFP (pNLENG1-ES-IRES-GFP) was described previously [35]. The HIV Bal.01 Env expression vector was obtained from the AIDS Research and Reference Reagent Program of the National Institute of Health. For single round HIV infection particle assembly, pHIV NL4-3-ΔEnv-eGFP and pHIV Bal.01-Env were co-transfected into HEK-293 T cells with the transfection reagent PEIpro^®^ (Polyplus transfection, New York, USA). For mouse CD24 reporter HIV particle assembly, pHIV R3A-HSA was transfected into HEK-293T cells with PEIpro^®^. Virus-containing supernatant was collected at 48 h and 72 h post-transfection. The supernatant harvested from transfected HEK-293T cells was first filtered through a 0.45 μm filter, and then purified and concentrated by centrifugation through a 10% sucrose solution (8000 rpm, 4 °C for 3 h).

### 2.4. MTS Assay

Cultured macrophages were incubated with or without imiquimod for 72 h in a humidified, 5% CO_2_ atmosphere. Into each 96-well assay plate containing the cells in 100 μL of culture medium, 20 μL of MTS reagent (Promega Co., Madison, WI, USA) was added, then the plate was incubated in darkness at 37 °C for 4 h. The absorbance at 490 nm was recorded by a 96-well plate reader (SpectraMax M5, Molecular Devices, San Jose, CA, USA).

### 2.5. Imiquimod Treatment and HIV Infection

Monocyte-derived macrophages were treated with or without imiquimod under three different treatment conditions (before, simultaneously with, and after infection). The cells were infected with an equal amount of cell-free HIV strains (Bal (RT = 2 × 10^5^), Jago (RT = 7.5 × 10^4^), or YU2 (RT = 1 × 10^5^) for 3 h at 37 °C. The cells were then washed three times with DMEM to remove input viruses and then RNAs and proteins were collected from the cells and the culture supernatant 9 days post-infection.

### 2.6. RNA Extraction and Real-Time Polymerase Chain Reaction

Total RNAs from macrophages or cell-free supernatant were extracted using TRI Reagent or TRI Reagent BD (Sigma-Aldrich, St. Louis, MO, USA) according to the manufacturer’s instructions. RNAs were subjected to a reverse transcription reaction by using the random primer, dNTPs, AMV reverse transcriptase, and RNasin^®^ ribonuclease inhibitor (Promega Co., Madison, WI, USA) to obtain cDNA. cDNAs were then used as templates for the real-time polymerase chain reaction (real-time PCR) which was performed with PowerUp SYBR Green Master Mix (Applied Biosystems, Carlsbad, CA, USA). The level of GAPDH mRNA was used as an endogenous reference to normalize the quantities of target mRNAs. The oligonucleotide primers used in this study were synthesized by Integrated DNA Technologies, Inc. (Coralville, IA, USA) and listed in Table 1.

### 2.7. Western Blot

Macrophages were lysed with RIPA buffer (Sigma-Aldrich, St. Louis, MO, USA) supplemented with a protease and phosphatase inhibitor cocktail (100×, Thermo Fisher Scientific, Waltham, MA, USA). Proteins from the culture supernatant were extracted by the TCA/acetone precipitation method. Briefly, 0.5 mL of culture supernatant was precipitated with 0.5 mL of 20% TCA at −20 °C for 1 h in an Eppendorf microcentrifuge tube. The mixture was centrifuged at 11,500 rpm for 15 min at 4 °C and then the supernatant was discarded. The pellet was washed with 1 mL of ice-cold acetone three times and the pellet was left to dry at room temperature. The pellet was lysed with Western blot lysis buffer. The concentration of proteins was determined by BCA assay (Thermo Fisher Scientific, Waltham, MA, USA). Equal amounts of proteins, along with protein molecular weight, were separated on 4 to 12% Bis-Tris gels (Invitrogen, Grand Island, NY, USA) and transferred to an Immun-Blot^®^ PVDF membrane (Bio-Rad, Hercules, CA, USA). The blots were blocked with 5% nonfat milk for 2 h at room temperature and then incubated with primary antibodies overnight at 4 °C. The blots were washed in PBS with 0.1% Tween 20 (PBST) four times and then incubated with HRP-conjugated second antibodies for 2 h at room temperature. The blots were developed with SuperSignal West Pico Chemiluminescent Substrate (Thermo Fisher Scientific, Waltham, MA, USA) after 4 washes with PBST. The blots were then exposed to an iBright 1500 imaging analyzer (Invitrogen, Carlsbad, CA, USA).

### 2.8. Detection of HIV Strong-Stop DNA

To further study whether imiquimod could block HIV entry, we examined the effect of imiquimod on the expression of HIV strong-stop DNA which is the first product of HIV reverse transcription, an indicator of HIV entry [36,37]. HIV Bal was treated with RNase Free DNase I at room temperature for 30 min before the infection of the macrophages. Total DNA from cells and viral DNA products were collected at 3 h post-infection with the QIAamp DNA Mini kit (Qiagen, Inc., Valencia, CA, USA). Strong-stop DNA was then quantified by the real-time PCR with HIV LTR RU/5 primers (Table 1).

### 2.9. Flow Cytometry

Macrophages were treated with imiquimod for 24 h prior to HIV R3A-HSA infection, infected macrophages were harvested at 72 h post-infection with Versene solution (0.48 mM EDTA). The cells were immediately washed with a cell staining buffer prior to staining with APC-anti-mouse CD24 (M1/69), and APC-isotype IgG antibody-stained cells were used as the negative control. Macrophages were treated with or without imiquimod for 24 h before being harvested with the Versene solution. The harvested cells were stained with PE mouse anti-human CD4 antibody and PE mouse anti-human CCR5 antibody. PE-isotype IgG antibody-stained cells were used as the negative control. The stained cells were measured by a FACSCanto II (BD Bioscience, San Jose, CA, USA) and analyzed using FlowJo software (Tree Star Inc., Ashland, OR, USA).

### 2.10. Measurement of Green Fluorescence

Macrophages were treated with or without imiquimod for 24 h prior to HIV NL4-3-ΔEnv-eGFP-Bal infection. Three days after infection, the cells were washed with 1×PBS and stained for nuclei with Hochest 33342 (Invitrogen, Carlsbad, CA, USA) for 15 min. The stained cells were then photographed under a confocal microscope (Nikon, A1R, Tokyo, Japan) for green fluorescence measurement.

### 2.11. ELISA

Cell-free culture supernatant was collected from macrophage cultures treated with or without imiquimod for 24 h. The CC chemokines (MIP-1α, MIP-1β, and RANTES), tumor necrosis factor-alpha (TNF-α), interleukin (IL)-6, IL-8, IL-1β, and IL-10 were measured with ELISA kits based on the manufacturer’s instructions (R&D Systems Inc., Minneapolis, MN, USA).

### 2.12. Data Statistical Analysis

Data were expressed as means ± standard deviations (SD) from at least three independent experiments. For comparison of the mean of two groups (treated versus untreated), statistical significance was measured by the Student’s *t*-test. If there were more than two groups, a one-way analysis of variance followed by the Newman-Keul’s test were used. All statistical analysis was performed using GraphPad Prism 5.0 Software (GraphPad Software Inc., San Diego, CA). Statistical significance was defined as *p* < 0.05, *p* < 0.01.

## 3. Results

### 3.1. Imiquimod Inhibits HIV Infection of Macrophages

**Figure 1:**
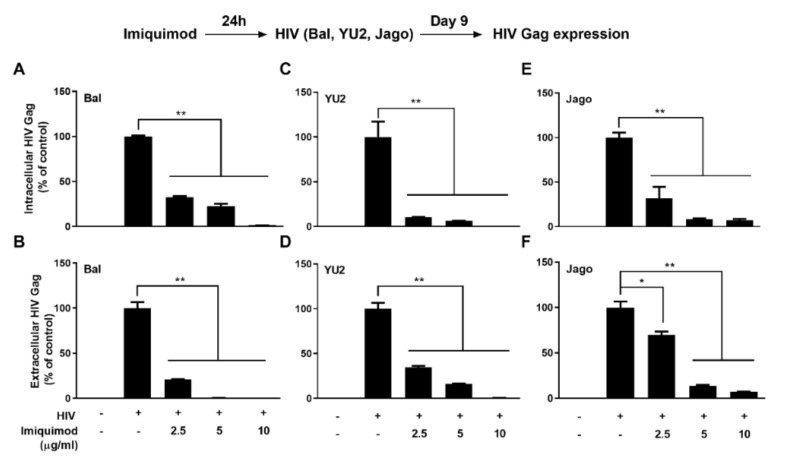
Imiquimod inhibits HIV Gag gene expression. (**A**–**F**) Macrophages were treated with or without imiquimod at indicated concentrations for 24 h prior to HIV (Bal, YU2, and Jago) infection. Cellular and culture supernatant RNAs were collected on day 9 post-infection and subjected to the real-time PCR for HIV Gag gene expression. Data are shown as mean ± SD of 3 independent experiments (* *p* < 0.05, ** *p* < 0.01).

**Figure 2:**
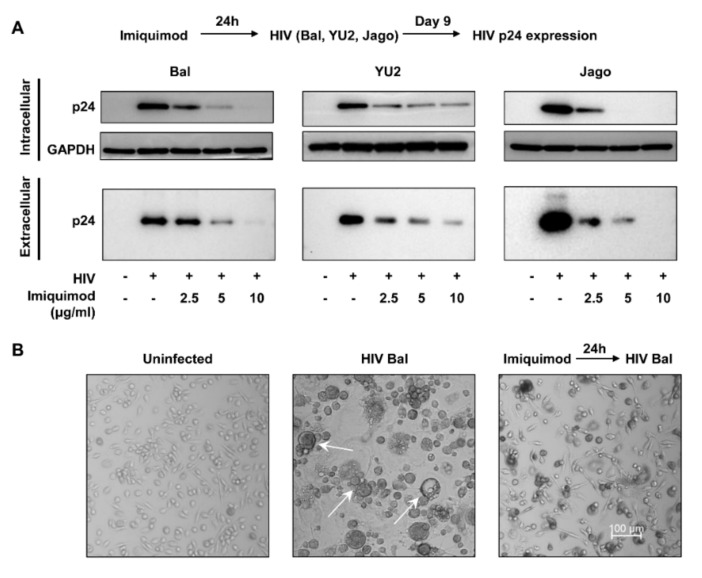
Imiquimod inhibits HIV p24 expression. (**A**) Macrophages were treated with or without imiquimod at indicated concentrations for 24 h prior to infection with different HIV strains (Bal, YU2, and Jago). Cellular and culture supernatant proteins were collected on day 9 post-infection and subjected to the Western blot assay for HIV p24 expression. (**B**) The morphology of uninfected macrophages comparing with HIV Bal-infected cells pretreated with or without imiquimod (10 μg/mL) was photographed by a confocal microscope (Nikon, A1R, Tokyo, Japan) on day 9 post-infection. The arrows indicate HIV-induced giant syncytium formation in macrophages.

To determine the effect of TLR7 activation by imiquimod on HIV infection in macrophages, cells were treated with or without imiquimod for 24 h prior to infection with different HIV strains (Bal, YU2, and Jago). On day 9 post-infection, we measured the mRNA expression of the HIV Gag gene and protein level of HIV p24 to evaluate the anti-viral effect of imiquimod. As shown in Figure 1, the imiquimod treatment of macrophages dose-dependently inhibited the HIV Gag gene expression in both cells (intracellular) and culture supernatant (extracellular). In addition, imiquimod significantly inhibited HIV p24 protein expression in macrophages and this inhibition was dose-dependent (Figure 2A). Morphologically, HIV Bal-infected macrophages showed characteristic giant syncytium formation (Figure 2B). However, imiquimod-treated macrophages failed to develop HIV-induced giant syncytia (Figure 2B).

**Figure 3:**
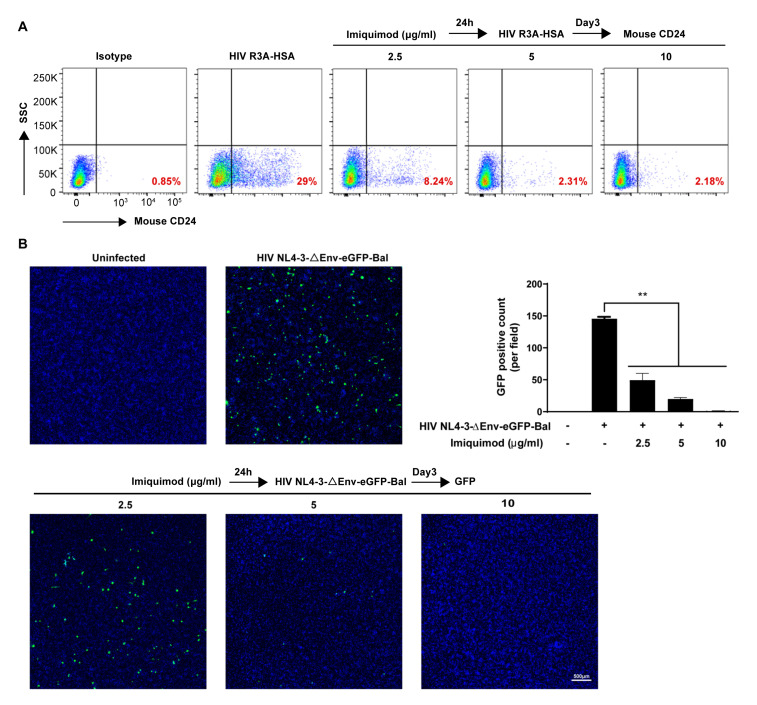
Imiquimod inhibits HIV R3A-HSA and HIV NL4-3-ΔEnv-eGFP-Bal infection. Macrophages were treated with or without imiquimod at the indicated concentrations for 24 h prior to infection. (**A**) Cells were washed 3 times to remove unattached HIV R3A-HSA viruses with DMEM at 4 h post-infection and then harvested cells at 72 h post-infection. The cells were then subjected to flow cytometry for mouse CD24 expression. (**B**) Cells were washed to remove unattached HIV NL4-3-ΔEnv-eGFP-Bal viruses with DMEM at 24 h post-infection. The green fluorescence protein expression was measured by confocal microscope (Nikon, A1R, Tokyo, Japan) at 72 h post-infection. Scale bar, 500 µm (** *p* < 0.01).

To visualize the effect of imiquimod on HIV, we performed the infection experiments with HIV R3A-HSA and HIV NL4-3-ΔEnv-eGFP-Bal. Macrophages were treated with or without imiquimod for 24 h prior to infection with the viruses. We measured the expression of mouse CD24 and GFP in the infected macrophages treated with or without imiquimod on day 3 post-infection. As shown in Figure 3A, imiquimod treatment of macrophages dose-dependently downregulated mouse CD24 expression. In addition, imiquimod-treated macrophages expressed significantly fewer GFP-positive cells in comparison with the untreated cells (Figure 3B).

To determine whether the inhibitory effect of imiquimod on HIV is not associated with cytotoxicity, we treated macrophages with different concentrations of imiquimod for 72 h and measured cell viability by MTS assay. As shown in Figure S1, imiquimod had little cytotoxic effect on macrophages at a dose as high as 160 μg/mL.

### 3.2. Imiquimod Blocks HIV Entry into Macrophages

**Figure 4:**
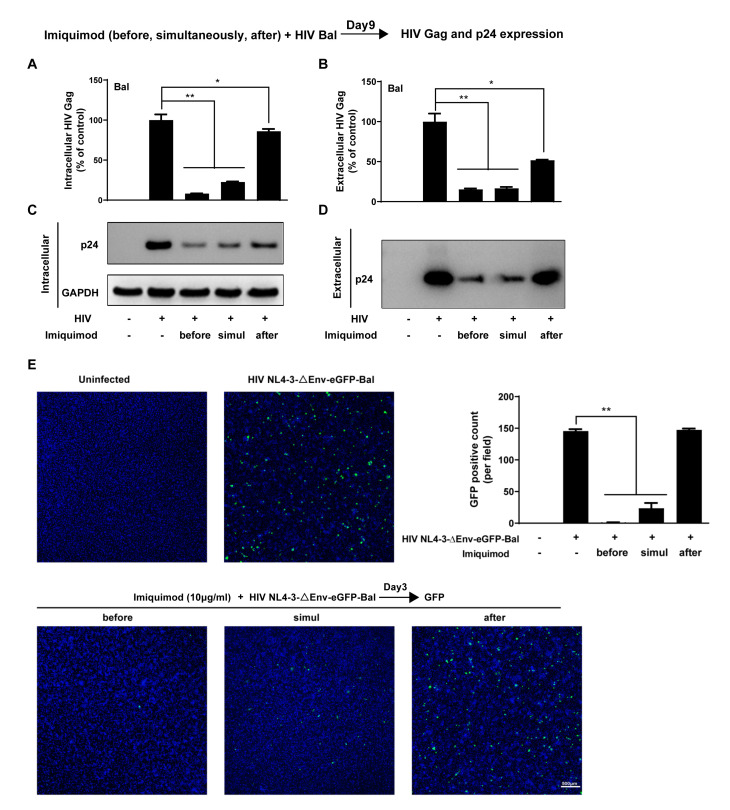
Imiquimod blocks HIV entry into macrophages. (**A**–**D**) Macrophages were treated with either imiquimod (10 μg/mL) for 24 h prior to HIV Bal infection (before), or imiquimod and infected with HIV Bal simultaneously (simul), or infected with HIV Bal for 3 h prior to imiquimod treatment (after). Cellular and culture supernatant RNAs were collected on day 9 post-infection and subjected to the real-time PCR for HIV Gag gene expression (**A**,**B**). Proteins of cells and culture supernatant were collected on day 9 post-infection and subjected to Western blot for HIV p24 expression (**C**,**D**). (**E**) Macrophages were treated with either imiquimod (10 μg/mL) for 24 h prior to HIV NL-4-3-ΔEnv-eGFP-Bal infection (before), or imiquimod and infected with HIV NL-4-3-ΔEnv-eGFP-Bal simultaneously (simul), or infected with HIV NL-4-3-ΔEnv-eGFP-Bal for 24 h prior to imiquimod treatment (after). At 72 h post-infection, the green fluorescence was measured by confocal microscope. Data are shown as mean ± SD of 3 independent experiments. Scale bar, 500 μm (* *p* < 0.05, ** *p* < 0.01).

To further determine imiquimod-mediated HIV inhibition, we also examined the anti-HIV effect of imiquimod under three different treatment conditions (before, simultaneously with, and after infection). Briefly, macrophages were treated with imiquimod for 24 h prior to HIV Bal infection (before), or cells were treated with imiquimod and infected with HIV Bal simultaneously (simul), or cells were first infected with HIV Bal for 3 h and then treated with imiquimod (after). Similarly, the mRNA expression of the HIV Gag gene and the protein expression of viral p24 were measured on day 9 post-infection. As shown in Figure 4, while imiquimod significantly inhibited the HIV Gag gene (Figure 4A,B) and p24 protein expression (Figure 4C,D) under all of the treatment conditions, the treatment of the cells with imiquimod before infection was the most effective in suppressing HIV. These similar findings were also observed in the HIV NL4-3-ΔEnv-eGFP-Bal infection experiments under the same imiquimod treatment conditions. As shown in Figure 4E, cells pretreated with imiquimod had the lowest level of GFP-positive cells, while treatment after infection had little impact on the GFP expression in the cells.

### 3.3. Imiquimod Induces CC Chemokines

**Figure 5:**
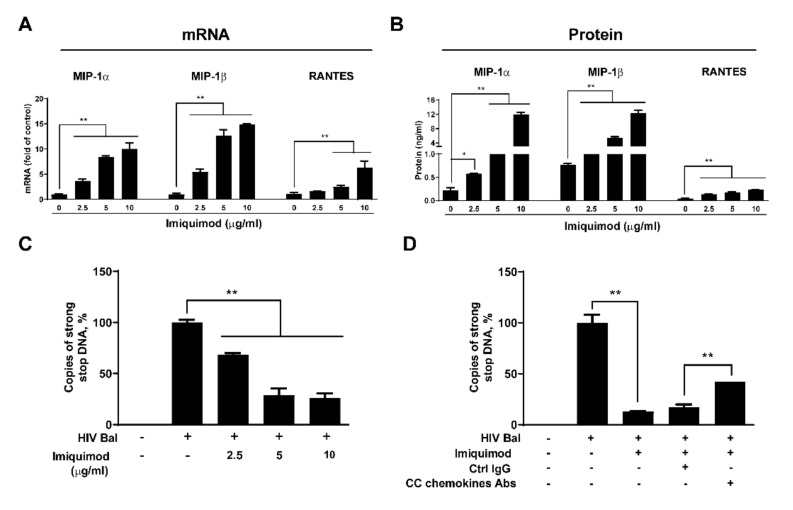
Imiquimod induces CC chemokines. (**A**,**B**) Macrophages were treated with or without imiquimod at the indicated concentrations. Total cellular RNA was extracted at 6 h post-treatment and subjected to the real-time PCR for the mRNA levels of MIP-1α, MIP-1β, RANTES, and GAPDH (**A**). Twenty-four hours after imiquimod treatment, culture supernatant was then collected and subjected to the ELISA assay for the protein levels of MIP-1α, MIP-1β, and RANTES (**B**). (**C**) HIV strong-stop DNA was detected in macrophages pretreated with imiquimod at the indicated concentrations. (**D**) Macrophages were pretreated with imiquimod (10 μg/mL) for 24 h and then were incubated with 20 μg/mL control IgG or a mixture of neutralization antibodies to MIP-1α, MIP-1β, and RANTES for 2 h prior to infection with HIV Bal which had been treated with RNase Free DNase I. HIV strong-stop DNA was quantified at 3 h post-infection. Data are shown as mean ± SD of three independent experiments (* *p* < 0.05, ** *p* < 0.01).

Based on the findings in the HIV infection experiments, we next studied the cellular factors associated with HIV entry into macrophages. It is well known that CC chemokines (MIP-1α, MIP-1β, and RANTES) are the natural ligands for the HIV co-receptor CCR5 and could block HIV entry [38]. We thus examined whether imiquimod can induce CC chemokines production in macrophages, and we found that the imiquimod treatment of macrophages dose-dependently induced the expression of all three CC chemokines at both mRNA and protein levels (Figure 5A,B). In addition, we found that imiquimod could block the expression of the strong-stop DNA in macrophages (Figure 5C), the first product of HIV reverse transcription [36,37]. The expression level of HIV strong-stop DNA from the imiquimod treated group was downregulated by ~1.5 folds to ~4 folds compared to the untreated group (Figure 5C). To determine the role of the CC chemokines (MIP-1α, MIP-1β, and RANTES) in imiquimod-mediated HIV inhibition, we used the neutralization antibodies to MIP-1α, MIP-1β, and RANTES in the blocking experiments. We observed that the neutralization antibodies to MIP-1α, MIP-1β, and RANTES could compromise the effect of imiquimod on the inhibition of HIV in macrophages (Figure 5D).

### 3.4. Imiquimod Induces Inflammatory Cytokines

**Figure 6:**
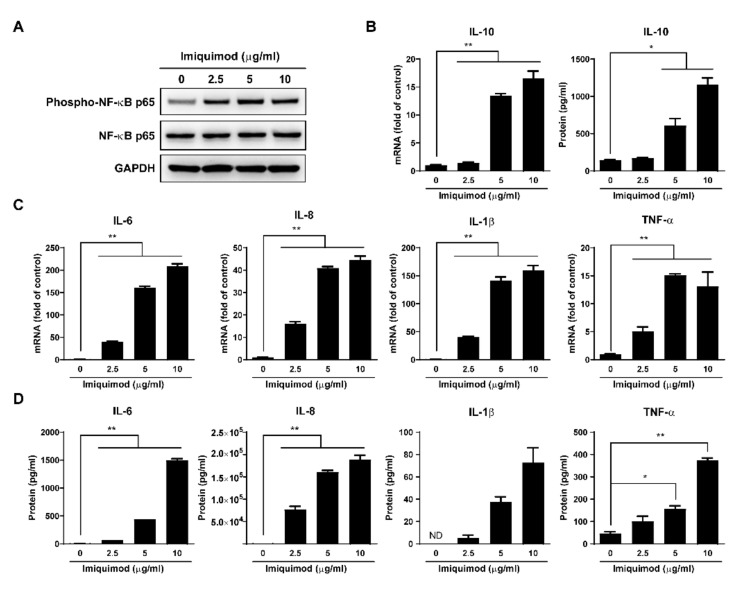
Effect of imiquimod on NF-κB signaling pathway. (**A**) Macrophages were treated with imiquimod at indicated concentrations for 24 h. Then cellular protein was collected and subjected to Western blot for GAPDH, NF-κB p65, and Phospho-NF-κB p65 expression. (**B**) Macrophages were treated with imiquimod at indicated doses for 6 h or 24 h, and then cellular RNA or culture supernatant was collected and subjected to real-time PCR or ELISA for IL-10 expression. (**C**) Macrophages were treated with imiquimod at indicated concentrations for 6 h. Then cellular RNA was collected and subjected to real-time PCR for IL-6, IL-8, IL-1β, and TNF-α expression. (**D**) Macrophages were treated with imiquimod at indicated concentrations for 24 h. Then culture supernatant was collected and subjected to ELISA for IL-6, IL-8, IL-1β, and TNF-α expression. Data are shown as mean ± SD of three independent experiments (ND: not detected, * *p* < 0.05, ** *p* < 0.01).

Because TLR7 activation could activate the nuclear factor kappa-B (NF-κB) signaling pathway [39,40], we investigated the effect of imiquimod on inflammatory cytokine expression in macrophages. We observed that although imiquimod had little effect on total NF-κB p65, it induced the phosphorylated NF-κB p65 expression in a dose-dependent manner (Figure 6A). As a result, there was a significant increase in the mRNA expression of inflammatory cytokines (IL-6, IL-8, IL-1β, and TNF-α) and anti-inflammatory cytokine (IL-10) in imiquimod-treated macrophages (Figure 6B,C). In addition, we also measured the protein levels in the supernatant of macrophage cultures. As shown in Figure 6B,D, imiquimod-treated macrophages produced significantly higher levels of IL-10, IL-6, IL-8, IL-1β, and TNF-α in comparison with untreated cells, and this upregulation was dose-dependent.

### 3.5. Imiquimod Suppresses CD4 and CCR5 Expression

**Figure 7:**
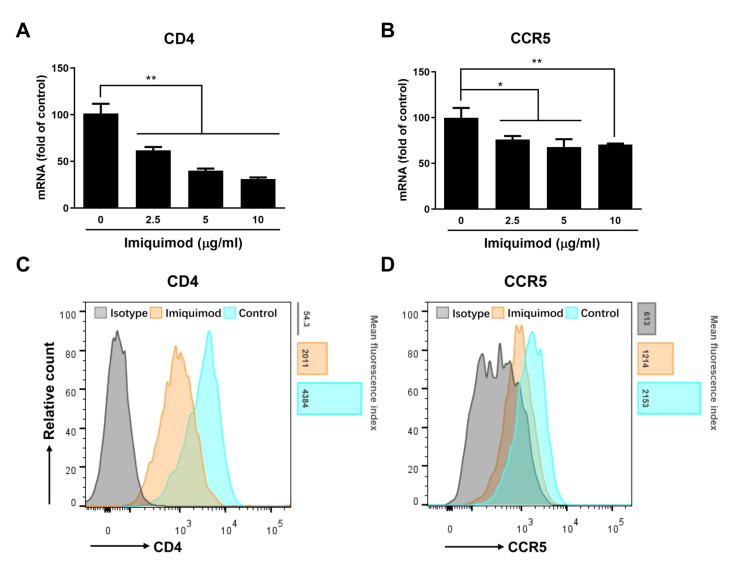
Effect of imiquimod on CD4 and CCR5. (**A**,**B**) Macrophages were treated with imiquimod at the indicated concentrations for 12 h. Cellular RNAs were collected and subjected to real-time PCR for CD4 and CCR5 mRNA expression. (**C**,**D**) Macrophages were treated with imiquimod (10 μg/mL) for 24 h and then collected for the flow cytometry analysis of CD4 and CCR5 protein expression. Data are shown in A and B as mean ± SD of three independent experiments (* *p* < 0.05, ** *p* < 0.01). Flow cytometry data shown in C and D are the representative pictures of two independent experiments.

HIV (R5-tropic strain) infection of macrophages requires two key entry receptors (CD4 and CCR5) on the cell surfaces. Therefore, we studied whether imiquimod has an impact on the expression of these receptors. As shown in Figure 7, imiquimod treatment of macrophages inhibited the expression of HIV entry receptors CD4/CCR5 at both mRNA (1.5 to 3.5 folds decrease) and protein (1.8 to 2.2 folds decrease) levels.

## 4. Discussion

TLR7 is highly expressed by plasmacytoid dendritic cells [41] and other immune cells including macrophages [42]. Agonists to TLR7 are attractive therapeutic agents with the potential of activating innate and acquired immunity against viral infections, including HIV [43,44,45]. In the present study, we demonstrated that TLR7 activation by imiquimod could effectively inhibit the infection of primary macrophages by different strains of HIV (Figure 1, Figure 2 and Figure 3). The anti-HIV properties associated with TLR7 activation have been documented by several groups using distinct small-molecule TLR7/8 agonists. Bam et al. [13] reported that the TLR7 agonist GS-9620 could potently inhibit the acute HIV infection of the human peripheral blood mononuclear cells (PBMC). However, they observed that GS-9620 had little effect on HIV in purified CD4^+^ T cells and macrophages. In contrast, Buitendijk et al., showed that in addition to HIV inhibition in activated PBMC, gardiquimod, another TLR7 agonist, could inhibit the HIV infection of macrophages when the cells were treated prior to or shortly after infection [12]. These conflicting findings could be due to the use of different TLR7 ligands and cell types in these studies. It should be noted that we used primary human macrophages derived from purified monocytes which were cultured in the absence of any growth factors such as granulocyte-macrophage colony-stimulating factor (GM-CSF). GM-CSF has been shown to have a contradictory role in the modulation of HIV infection [46].

To determine the mechanism(s) involved in imiquimod’s action on HIV infection of macrophages, we first examined the effect of imiquimod on the cytokines (IL-6, IL-8, IL-1β, IL-10, and TNF-α), including IFNs. We found that imiquimod could induce the phosphorylation of NF-κB p65 and the production of inflammatory cytokines (Figure 6), which agrees with the studies by others [16,39,40,47,48,49,50]. Several inflammatory cytokines are known to modulate HIV infection/replication in vitro, with their effects being either stimulatory (TNF-α, IL-1β, IL-6, and IL-8) or inhibitory (IFN-α and IL-10). As a potent anti-inflammatory cytokine, IL-10 can counteract the adverse effects of pro-inflammatory cytokines on HIV infection and inhibit HIV replication [51]. Therefore, it is likely that imiquimod-induced IL-10 compromises the stimulatory effects of the pro-inflammatory cytokines (TNF-α, IL-1β, IL-6, and IL-8) on HIV infection/replication in macrophages. In addition to the cytokines described above, we also examined the impact of imiquimod on the expression of the antiviral cytokine IFNs. Several studies demonstrated that the TLR7 agonists (GS-9620, gardiquimod, and resiquimod) could inhibit HIV replication in vitro [11,12,13] by inducing IFNs. The studies concluded that the induction of IFN-α is largely responsible for the TLR7 agonist-mediated anti-HIV effect. Interestingly, we observed that imiquimod did not significantly induce the expression of type I IFNs in macrophages (Figure S2). These conflicting findings could be due to differences in cell types and culture conditions. In this study, we used primary human macrophages derived from purified monocytes cultured in the absence of the growth factor, GM-CSF.

We hypothesized that imiquimod blocked HIV infection at the viral entry level based on the following observations: 1. Imiquimod treatment of macrophages before infection was the most effective in HIV inhibition (Figure 4); 2. Imiquimod significantly inhibited the expression of HIV strong-stop DNA (Figure 5C), the first synthetic (minus) strand DNA from viral RNA; 3. Imiquimod induced TNF-α which is known to target the HIV entry step specifically in macrophages [52]. TNF-α inhibits the entry of HIV into macrophages through downregulating CD4 and CCR5 receptors on the cell surface [53,54]. In addition, TNF-α can induce the expression of CC chemokines (MIP-1α, MIP-1β, and RANTES) [55,56], the ligands for CCR5. Therefore, we examined the impact of imiquimod on the HIV entry cellular factor (CD4, CCR5, and CC chemokines) and we found that imiquimod treatment of macrophages induced expression of the CC chemokines (MIP-1α, MIP-1β, and RANTES) at both mRNA and protein levels (Figure 5A,B). Imiquimod also inhibited the expression of CD4 and CCR5 receptors (Figure 7). Importantly, the role of CC chemokines in imiquimod-mediated HIV inhibition was confirmed by the finding that the addition of antibodies to the CC chemokines in the macrophage cultures largely blocked the effect of imiquimod on HIV inhibition (Figure 5D).

**Figure 8:**
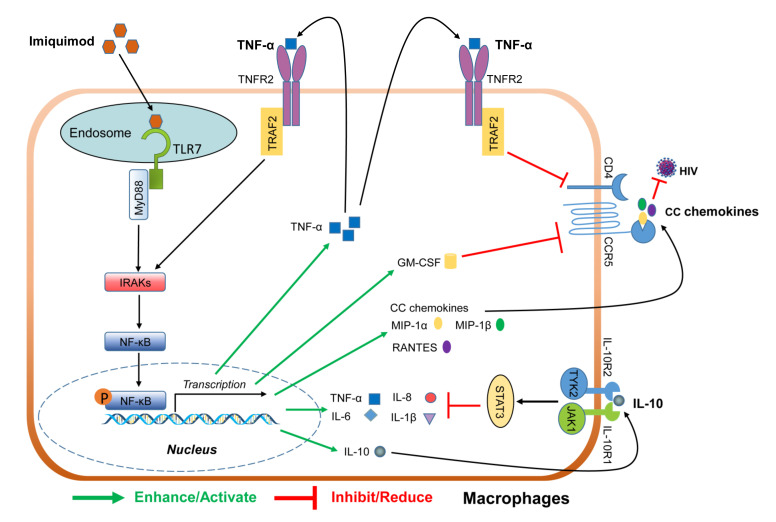
Schematic diagram of mechanisms for imiquimod-mediated HIV inhibition in macrophages. Through the activation of the NF-κB signaling pathway by binding to TLR7, imiquimod induces CC chemokines (MIP-1α, MIP-1β, and RANTES), TNF-α, IL-6, IL-1β, IL-8, and IL-10. As the natural ligands for HIV entry co-receptor CCR5, the CC chemokines can inhibit HIV entry into macrophages. Through binding to TNF receptor 2 (TNFR2), TNF-α inhibits the CD4 and CCR5 expression. In addition, TNF-α can induce the CC chemokines through binding to TNFR2 and activating the NF-κB signaling pathway. These combined activities result in the blocking of HIV entry into macrophages by imiquimod. As a potent anti-inflammatory cytokine, IL-10 can counteract the adverse effect of the pro-inflammatory cytokines on HIV infection and inhibit HIV replication. Therefore, it is likely that imiquimod-induced IL-10 compromises the stimulatory effects of the pro-inflammatory cytokines (TNF-α, IL-6, IL-1β, and IL-8) on HIV infection/replication in macrophages.

In summary, our study, for the first time, provides compelling experimental evidence that TLR7 activation by imiquimod significantly suppresses HIV infection through regulating the viral entry cellular factors in macrophages. Imiquimod treatment of macrophages could activate the NF-κB signaling pathway and induce multiple pro-inflammatory (TNF-α, IL-1β, IL-6, and IL-8) and anti-inflammatory (IL-10) cytokines. Importantly, imiquimod could induce the production of the CC chemokines which can competitively bind to CCR5 and block HIV entry into macrophages. In addition, imiquimod downregulates the expression of CD4 and CCR5 (Figure 8). While the precise cellular and molecular mechanisms for imiquimod-mediated HIV inhibition remain to be determined, the induction of CC chemokines and the downregulation of CD4 and CCR5 should be largely responsible for much of the imiquimod-mediated anti-HIV activity in macrophages. These findings are clinically important as they indicate that activating the intracellular antiviral immunity by imiquimod has the potential for HIV treatment. However, future ex vivo and in vivo investigations with animal models and clinical specimens are necessary, not only for confirming our in vitro findings, but also for developing a TLR7 agonist-based therapy for HIV disease.

## Figures and Tables

**Table 1:** Primer sets for real-time PCR.

Primer	Accession No.	Orientation	Sequences	Product (bp)
GAPDH	NM_002046	Sense Antisense	5′-GGTGGTCTCCTCTGACTTCAACA-3′ 5′-GTTGCTGTAGCCAAATTCGTTGT-3′	127
GAG	NC_001802.1	Sense Antisense	5′-ATAATCCACCTATCCCAGTAGGAGAAA-3′ 5′-TTTGGTCCTTGTCTTATGTCCAGAATGC-3′	115
CD4	NM_001382706.1	Sense Antisense	5′-AGTCCCTTTTAGGCACTTGC-3′ 5′-GATCATTCAGCTTGGATGG-3′	224
CCR5	NM_001100168.2	Sense Antisense	5′-CAAGTGTCAAGTCCAATCTA-3′ 5′-ACCAAAGATGAACACCAGTG-3′	123
MIP-1α	NM_021006.5	Sense Antisense	5′-GCTGACTACTTTGAGACGAGC-3′ 5′-CCAGTCCATAGAAGAGGTAGC-3′	252
MIP-1β	NM_002984.4	Sense Antisense	5′- CCAAACCAAAAGAAGCAAGC -3′ 5′- AGAAACAGTGACAGTGGACC -3′	314
RANTES	NM_002985.3	Sense Antisense	5′- CTGCATCTGCCTCCCCATA -3′ 5′- GCGGGCAATGTAGGCAAA -3′	62
HIV-1 LTR RU/5	NC_001802.1	Sense Antisense	5′-TCTCTCTGGTTAGACCAGATCTG-3′ 5′-ACTGCTAGAGATTTTCCACACTG-3′	180
IL-6	NM_001371096	Sense Antisense	5′-AGGAGACTTGCCTGGTGAAA-3′ 5′-CAGGGGTGGTTATTGCATCT-3′	180
IL-8	NM_000584	Sense Antisense	5′-ATGACTTCCAAGCTGGCCGTGGCT-3′ 5′-TCTCAGCCCTCTTCAAAAACTTCTC-3′	292
IL-1β	NM_000576	Sense Antisense	5′-AGGTGCATCGTGCACATAAG-3′ 5′-AAGCTGATGGCCCTAAACAG-3′	281
TNF-α	NM_000594	Sense Antisense	5′-CGAGTGACAAGCCTGTAGC-3′ 5′-GGTGTGGGTGAGGAGCACAT-3′	215
IL-10	NM_001382624	Sense Antisense	5′-CTTTAATAAGCTCCAAGAGAAAGGC-3′ 5′-CAGATCCGATTTTGGAGACC-3′	167
IFN-α	NM_002169.3	Sense Antisense	5′-TTTCTCCTGCCTGAAGAACAG-3′ 5′-GCTCATGATTTCTGCTCTGACA-3′	373
IFN-β	NM_002176.4	Sense Antisense	5′-GCCGCATTGACCATCTATGAGA-3′ 5′-GAGATCTTCAGTTTCGGAGGTAAC-3′	346

## Data Availability

Not applicable.

## Supplementary Materials

The following are available online at https://www.mdpi.com/article/10.3390/biology10070661/s1, Figure S1: Effect of Imiquimod on cell viability of macrophages, Figure S2: Effect of Imiquimod on IFNs expression, File S1: Original images of blots.
