# Supplementary material for: TLR7 Activation of Macrophages by Imiquimod Inhibits HIV Infection through Modulation of Viral Entry Cellular Factors
Source: Biology (Basel). 2021 Jul 13;10(7):661. doi: 10.3390/biology10070661 (PMC8301371; doi:10.3390/biology10070661)
Supplement: Supplementary file 1 [file biology-10-00661-s001.zip › original images of blots.pptx]

## Slide 1
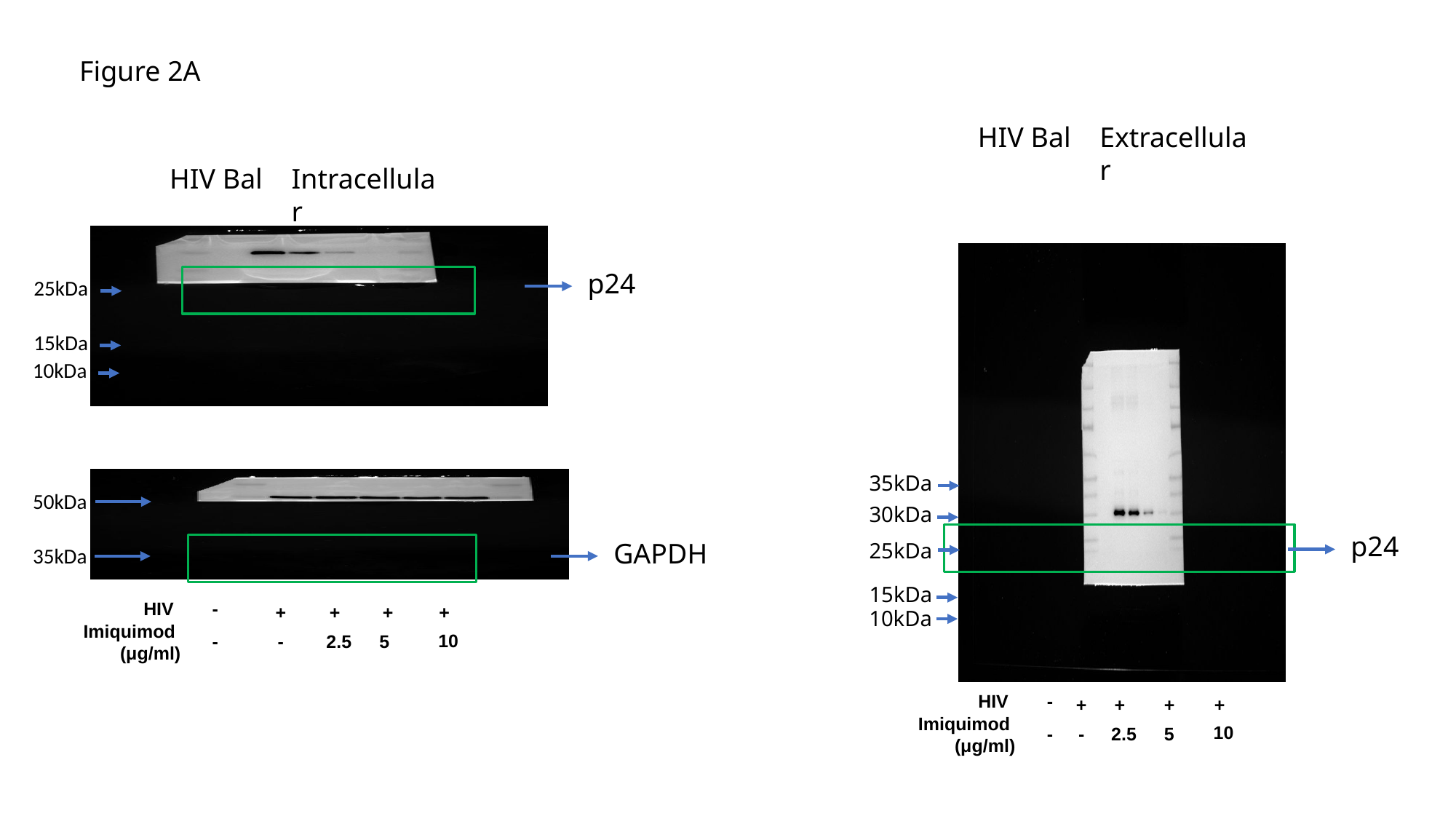

Figure 2A
HIV Bal
Extracellular
HIV Bal
Intracellular
p24
25kDa
15kDa
10kDa
35kDa
50kDa
30kDa
p24
GAPDH
25kDa
35kDa
15kDa
HIV
-
+
+
+
+
10kDa
Imiquimod
(μg/ml)
10
-
-
2.5
5
HIV
-
+
+
+
+
Imiquimod
(μg/ml)
10
-
-
2.5
5

## Slide 2
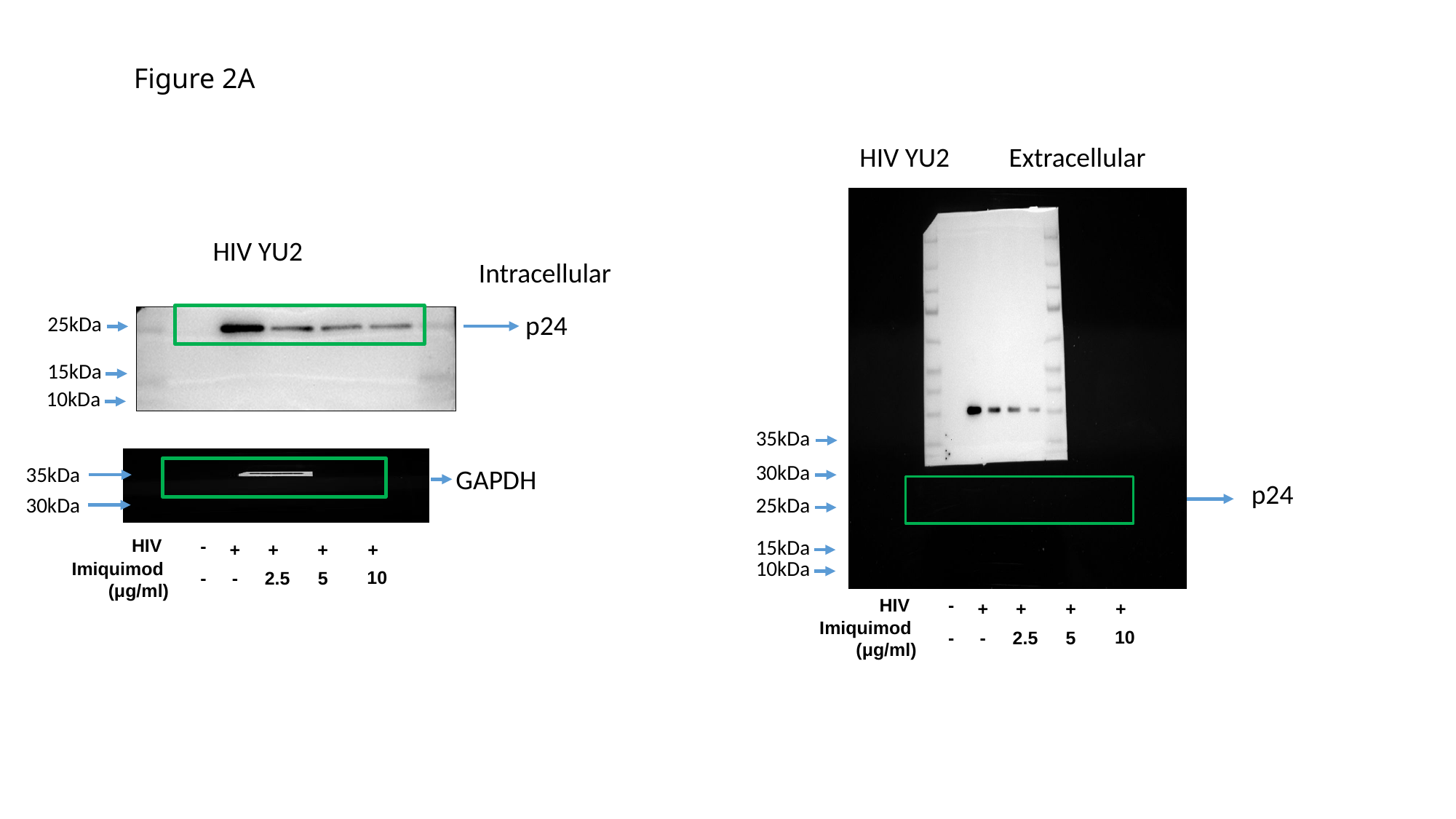

Figure 2A
HIV YU2
Extracellular
HIV YU2
Intracellular
p24
25kDa
15kDa
10kDa
35kDa
30kDa
35kDa
GAPDH
p24
30kDa
25kDa
15kDa
HIV
-
+
+
+
+
10kDa
Imiquimod
(μg/ml)
10
-
-
2.5
5
HIV
-
+
+
+
+
Imiquimod
(μg/ml)
10
-
-
2.5
5

## Slide 3
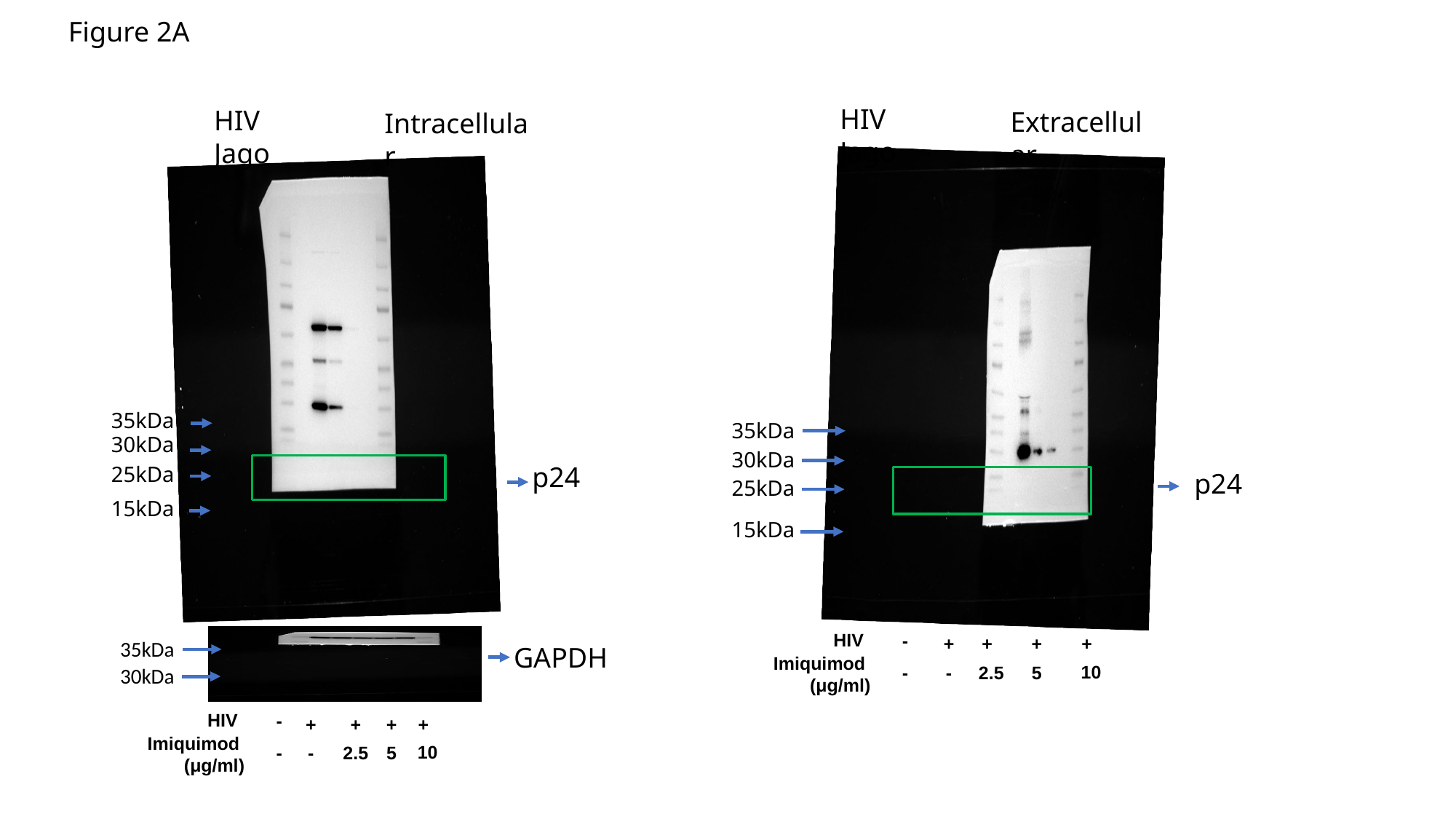

Figure 2A
HIV Jago
HIV Jago
Extracellular
Intracellular
35kDa
35kDa
30kDa
30kDa
p24
25kDa
p24
25kDa
15kDa
15kDa
HIV
-
+
+
+
+
35kDa
GAPDH
Imiquimod
(μg/ml)
10
-
-
2.5
5
30kDa
HIV
-
+
+
+
+
Imiquimod
(μg/ml)
10
-
-
2.5
5

## Slide 4
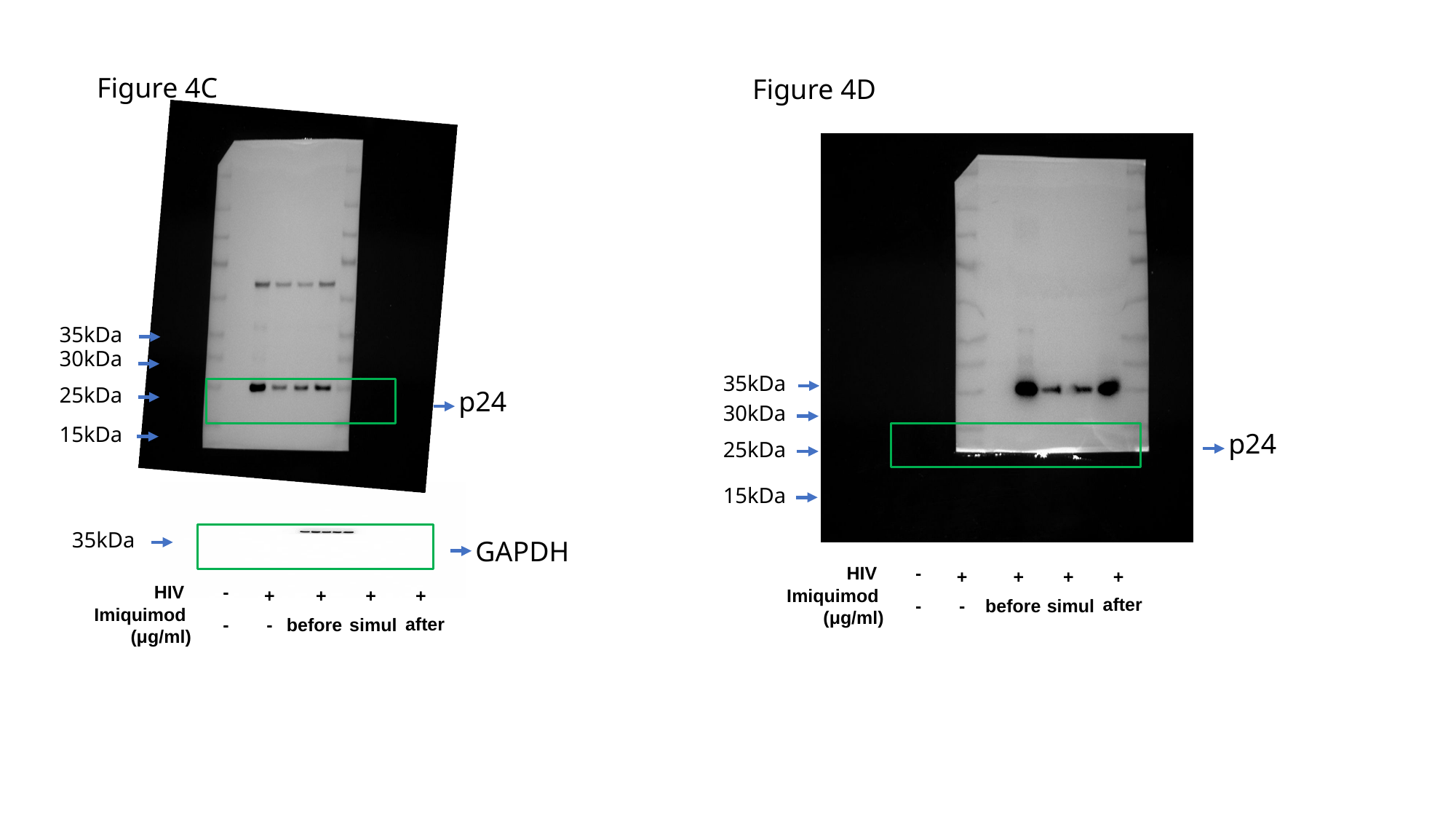

Figure 4C
Figure 4D
35kDa
30kDa
35kDa
25kDa
p24
30kDa
15kDa
p24
25kDa
15kDa
35kDa
GAPDH
HIV
-
+
+
+
+
HIV
-
Imiquimod
(μg/ml)
+
+
+
+
after
-
-
before
simul
Imiquimod
(μg/ml)
after
-
-
before
simul

## Slide 5
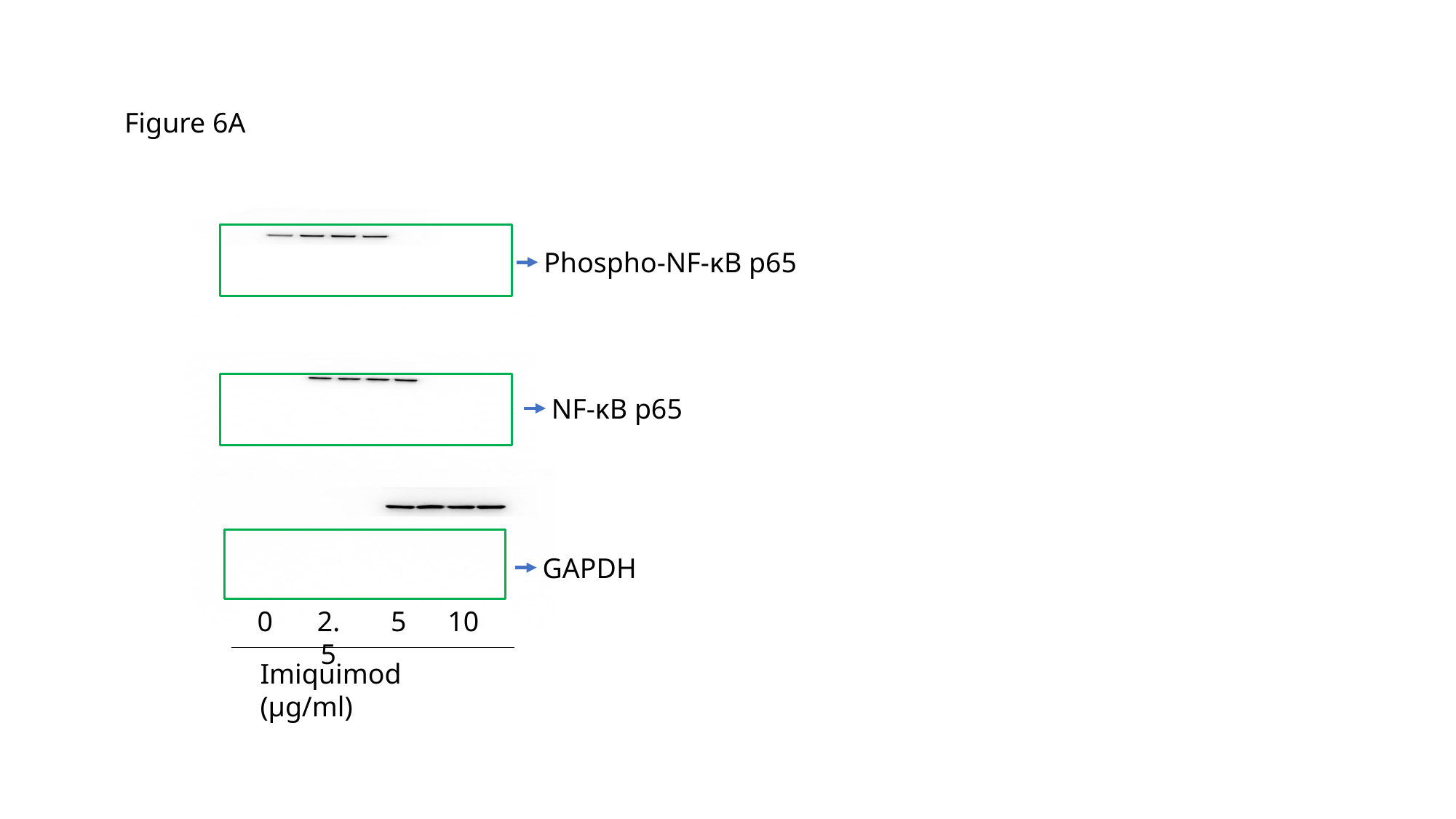

Figure 6A
Phospho-NF-κB p65
NF-κB p65
GAPDH
0
2.5
5
10
Imiquimod (μg/ml)
