# Supplementary figures and images for: TLR7 Activation of Macrophages by Imiquimod Inhibits HIV Infection through Modulation of Viral Entry Cellular Factors
Source: Biology (Basel). 2021 Jul 13;10(7):661. doi: 10.3390/biology10070661 (PMC8301371; doi:10.3390/biology10070661)

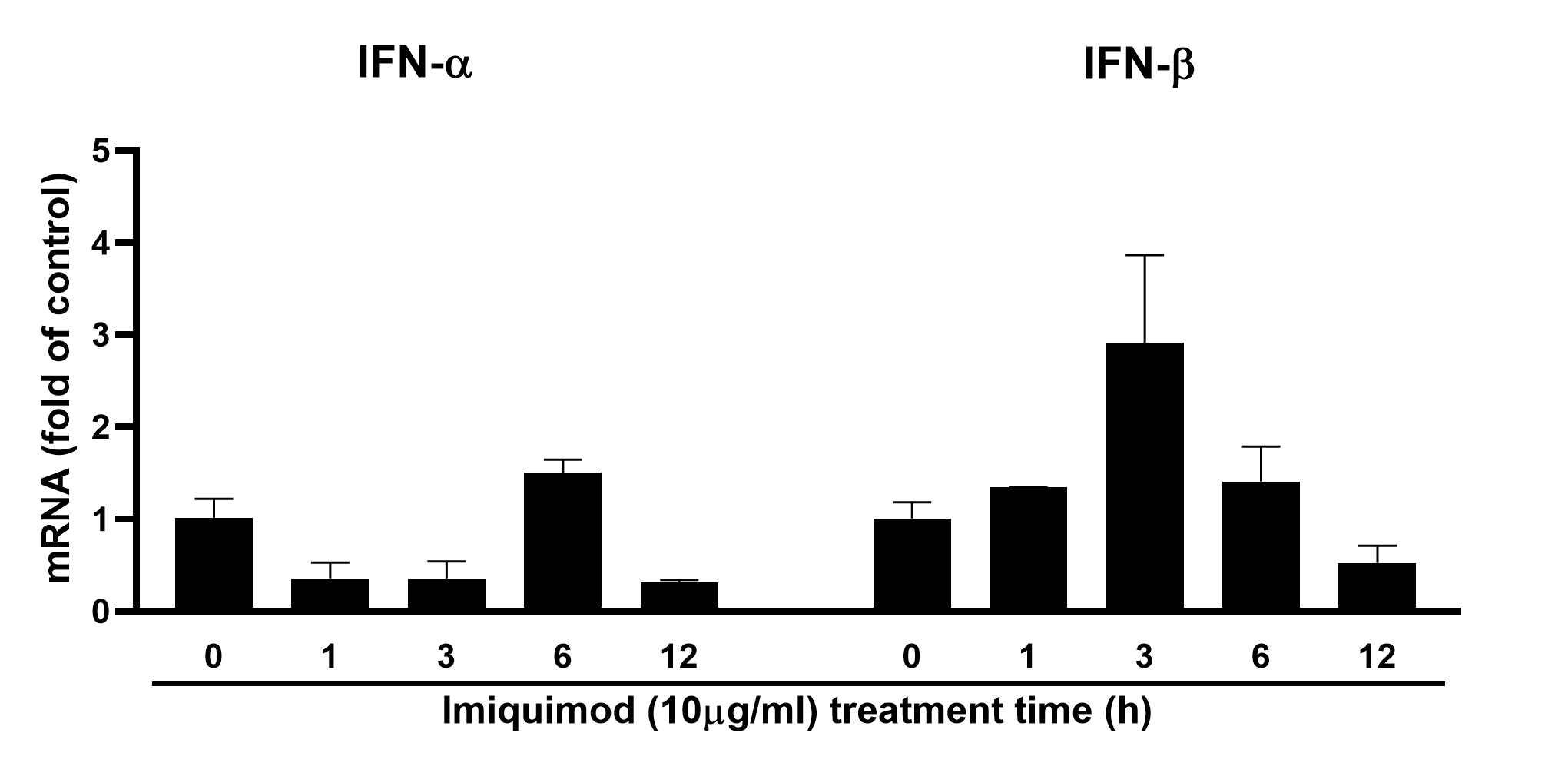

Supplement: Supplementary file 1 [file biology-10-00661-s001.zip › Figure S2.tif]
